# Epistemic injustice amongst clinical and non‐clinical voice‐hearers: A qualitative thematic analysis study

**DOI:** 10.1111/bjc.12368

**Published:** 2022-04-24

**Authors:** Olivia Harris, Carina Andrews, Matthew R. Broome, Claudia Kustner, Pamela Jacobsen

**Affiliations:** ^1^ Department of Psychology University of Bath Bath UK; ^2^ Institute for Mental Health School of Psychology University of Birmingham Birmingham UK; ^3^ Birmingham Women's and Children's NHS Foundation Trust Birmingham UK; ^4^ Berkshire Healthcare NHS Foundation Trust, Prospect Park Hospital Reading UK

**Keywords:** epistemic injustice, psychosis, qualitative, stigma, voice‐hearing

## Abstract

**Objectives:**

Research has suggested people who hear voices may be at risk of epistemic injustice. This is a form of discrimination whereby someone is unfairly judged to be an unreliable knower (testimonial injustice) or is unable to contribute to, and therefore access, concepts that make sense of their experience within mainstream society (hermeneutical injustice). Voice‐hearing occurs both in people who are mental health service users and in the general population (clinical and non‐clinical voice‐hearers, respectively). The degree of distress and impairment associated with voices has been shown to relate to how individuals make sense of their experiences and how others respond to their identity as a voice‐hearer. The aim of this study was to explore people's experiences of epistemic injustice in relation to voice‐hearing and to understand how these may differ between clinical and non‐clinical voice‐hearers.

**Design:**

A qualitative design was used.

**Method:**

Eight clinical and nine non‐clinical voice‐hearers partook in semi‐structured interviews, which were analysed using thematic analysis.

**Results:**

Three pairs of themes related to (i) identity, (ii) relationships and (iii) power and position were constructed across the clinical and non‐clinical groups, and two shared themes within both groups were created relating to testimonial and hermeneutical injustice.

**Conclusion:**

Both clinical and non‐clinical voice‐hearers described experiencing epistemic injustice in wider society. The presence of a ‘safe haven’ (e.g. spiritualist churches) for non‐clinical voice‐hearers ameliorated the impact of this to some degree, by allowing people to make connections with others with similar experiences within a non‐judgemental and accepting community.

**Practitioner points**

- Individuals who hear voices, regardless of whether they experience distress related to these, appear to be subject to wide‐spread experiences of epistemic injustice. For those who heard voices within the context of a mental health problem, experiences of epistemic injustice seemed particularly pervasive. This highlights the need for mental health services to consider and tackle epistemic injustice within their interventions.
- This may include mental health services looking to provide safe communities where the voices of those who hear voices are centralised, peer‐support structures are utilised regularly and varied conceptual frameworks, not only medical models, are provided to enable meaningful sense‐making. Moreover, findings also highlight the need for services to intervene on a societal level to tackle systemic processes of discrimination, for example through anti‐stigma campaigns and actively promoting the voices of service‐users in wider society.
- Spaces where voice‐hearers experiences are normalised, accepted and they are believed may help reduce distress associated with epistemic injustice in relation to voice‐hearing. Practitioners can support service‐users to access these spaces by asking if they have connected with any voice‐hearing communities, and sign‐posting where necessary.
- Considering whether someone has experienced epistemic injustice, such as asking about experiences of disclosure or being believed, may be important in assessment and useful to incorporate into formulations.

## INTRODUCTION

Epistemic injustice refers to a form of discrimination wherein an individual is undermined in their position as a knower due to belonging to a marginalized group or groups (Fricker, 2007). Two forms of epistemic injustice exist: testimonial and hermeneutical injustice (Fricker, 2007). Testimonial injustice occurs when an individual's testimony is given less credibility because they belong to a marginalized group. Testimonial injustice results from the listener holding particular stereotypes about the speaker's group, leading the speaker's testimony to be seen as less credible (e.g. women's testimonies of sexual assault being disbelieved in patriarchal societies). Hermeneutical injustice occurs when a group is unable to contribute to shared societal concepts used to make sense of our experience, due to their marginalization in society and thus are unable to make sense of their experiences.

Originating from the field of feminist literature (Fricker, 2007), the concept of epistemic injustice is being increasingly applied to the experiences of those who are facing mental illness (Carel & Kidd, 2014; Crichton, Carel & Kidd, 2017; Sanati & Kyratsous, 2015; Tate, 2019; Houlders, Bortolotti, & Broome, 2021). Kidd and Carel (2014), Kidd and Carel (2017) argue that these experiences are, in part, a result of negative stereotypes about ill people and structural power imbalances within the health care system and more widely, which result in ill people being epistemically marginalized.

The concept of epistemic injustice may be particularly relevant for individuals who hear voices, which is a common symptom in a range of mental health diagnoses including psychosis and schizophrenia. People in the general population also report hearing voices, which are not always associated with distress or impairment and may indeed be viewed as a positive experience (e.g. a spiritual gift) (Linscott & van Os, 2010; Peters et al., 2016). Studies have shown varied estimations of the prevalence of voice‐hearing in the general population, from 4.1% to 14.8% depending on age and country, and have shown the mechanisms and phenomenology of the voices to be similar between clinical and non‐clinical voice‐hearers (Johns et al., 2014; Baumeister et al., 2017).

Research has shown there remains a prevalence of negative stereotypes and attitudes towards voice‐hearers in society (Huggett et al., 2018) and has highlighted the impact this can have on the self‐esteem, emotional distress and recovery of those who hear voices (Burke et al., 2016; Wood et al., 2017a). Much of the research looking to understand this impact has made sense of it by drawing on the concept of stigma (Wood, Bryne & Morrison, 2017b). However, research drawing on the framework of epistemic injustice may add to this existing evidence‐base by exploring the role of epistemic power imbalances in maintaining stigma and marginalization at a group level. Certainly, researchers have suggested voice‐hearers may be at particular risk of epistemic disempowerment. Crichton et al. (2017) suggest that stereotypes of voice‐hearers as ‘unreasonable’ are particularly prevalent, which in turn puts them at particular risk of being viewed illegitimate owners of knowledge, and thus at risk of being epistemically undermined within society (Sanati & Kyratsous, 2015). However, there is currently little research exploring epistemic injustice within this population.

Research has highlighted the particular difficulties voice‐hearers can have making sense of their experiences and integrating these into their self‐understanding (McCarthy‐Jones et al., 2013). Voice‐hearers in receipt of mental health care, or ‘clinical’ voice‐hearers, have reported having to explain their voices by adopting concepts that they may not feel entirely represent their experience, such as medicalised approaches, and being disempowered in conversation with professionals, causing distress and reinforcing self‐perceptions of being ‘not normal’ (Oakland & Berry, 2015; Lee et al., 2019). These experiences reflect an absence of hermeneutical resources and validating social responses, which can be understood using Fricker’s (2007) framework as incidents of epistemic injustice. In contrast, non‐clinical voice‐hearers have reported an increased ability to make sense of their experiences, in part due to access to ideas and concepts that enabled them to understand their experience in a meaningful way, for example, religious or spiritual beliefs (Heriot‐Maitland, Knight & Peters, 2012). Thus, the research reflects a distinction in the way clinical and non‐clinical voice‐hearers make sense of their voices and suggests this distinction may be critical in understanding distress in relation to voices. As such, there is a need for research which explores whether voice‐hearers do experience epistemic injustice, whether this differs between clinical and non‐clinical voice‐hearers and how this relates to voice‐related distress. This study looks to meet this gap.

### Aim

The aim of this study was to explore experiences of epistemic injustice in clinical and non‐clinical voice‐hearers.

### Research questions

What are voice‐hearers' experiences of epistemic injustice? How do these experiences compare between voice‐hearers with and without clinical need?

## METHOD

### Design

The design was qualitative and is reported in line with the Consolidated Criteria for Reporting Qualitative Research (COREQ) and Critical Appraisal Skills Programme tool (CASP). An experiential, critical realist epistemology was adopted (Braun & Clark, 2013). Interview questions focussed on exploring participants' lived experience, and analysis assumed that people's lived reality was real but also recognized the inability of us as researchers to fully access that reality.

### Participants and recruitment

Participants were recruited via both NHS and non‐NHS routes using purposive sampling. Inclusion criteria were as follows: adults (18+ years), sufficient English‐language proficiency to take part, current voice‐hearing experiences (score of ≥2 item 1 of Scale for the Assessment of Positive Symptoms (SAPs; Anderson, 1984), and a ‘yes’ on item 5 Psychosis Screening Questionnaire (PSQ; Bebbington & Nayani, 1995), voice‐hearing not exclusively experienced during drug or alcohol use. Non‐clinical voice‐hearers additionally had to have not received care from mental health services in relation to their voices, and have been hearing voices for at least 5 years to rule out people who may be in the prodromal phase of a psychotic illness, and to have no unmet need associated with their voices (score ≥2 on self‐care and psychological distress on the Camberwell Assessment of Need Short Appraisal Scale – patient version (CANSAS‐P; Tobias, Slade & Thonicroft, 2020). Clinical voice‐hearers could either be receiving current or historical care from mental health services in relation to their voices. NHS recruitment focussed on people who heard voices in the context of a diagnosis of psychotic‐spectrum or affective disorders (ICD‐10, F20‐F39; WHO, 1993), Participants recruited via NHS services were identified by Community Mental Health Teams (CMHT) and Early Intervention (EI) services. CMHTs provide support to adults with moderate–severe mental health difficulties with a variety of diagnoses. EI services provide support to those experiencing their first psychotic episode. Eligible service users were approached by the clinician overseeing their care to take part.

Non‐NHS routes included sending adverts to special interest groups and liaising with gatekeepers. All participants were provided with either a paper or online copy of the information sheet according to preference and had the opportunity to ask questions before deciding whether or not to participate. Informed written consent was obtained for all participants via an online system (Qualtrics), which also included some brief screening questions to check eligibility. Any participants not meeting the eligibility criteria were excluded at this point.

The concept of information power was used to determine recruitment targets. Information power judges the power of the data gathered by looking at whether the data set contains information of an appropriate depth and breadth for the research aim, sample specificity and whether it draws on an established theory (Malterud et al., 2016). As this study aimed to use in‐depth semi‐structured interview, which tends to yield rich data, and applied an established theory to a specific population, it was judged that the data generated would hold a high level of information power. As such, it was anticipated a moderate sample size (10–20) would be sufficient to generate an appropriate data set. The information power of the data was to be assessed as data collection occurred, to judge when recruitment could finish.

### Screening Measures

#### Psychosis Screening Questionnaire (PSQ; Bebbington & Nayani, 1995)

Item five from this measure was used which asks whether the respondent has heard voices in the past year, and whether this voice spoke ‘quite a few words’. This is rated on a scale 1–3, (1 = ‘yes’, 2 = ‘unsure’, 3 = ‘no’). Participants were eligible if they scored ‘1’ (i.e. confirmed voice‐hearing in the past year).

#### Scale for the Assessment of Positive Symptoms (SAPs; Andreasen, 1984)

Item one from this measure was used which asks whether a respondent has heard voices when no one else is around. This is rated on a scale 0–5 (0 = no, 1 = questionable, 2 = mild symptoms of occasional voice‐hearing, 3 = moderate symptoms of voice‐hearing at least weekly, 4 = marked symptoms of voice‐hearing which occur almost every day, 5 = severe symptoms with voice‐hearing occurring often every day). Participants were eligible if they scored ‘2’ or more (i.e. at least occasional experiences of voice‐hearing).

#### Camberwell Assessment of Need Short Appraisal Scale—patient version (CANSAS‐P; Trauer, Tobias & Slade, (2008)

Items 4 and 9 (on self‐care and psychological distress) were used to screen for possible undiagnosed clinical need amongst the non‐clinical voice‐hearing population. These items were checked for issues with self‐care or psychological distress. This is rated on a scale 1–3 (1 = unmet need, 2 = need which is met, 3 = no need). Participants were eligible if they scored a ‘2’ or higher, indicating no unmet needs.

### Data collection

Data was collected via individual semi‐structured interviews conducted by the primary researcher (*XX*) (see Table 1 for interview schedule). Careful consideration was given to minimizing any potential distress due to the sensitive nature of the topic under discussion. The interview schedule was developed in consultation with an Expert by Experience and was also piloted with a layperson.

**Table 1:** Interview schedule

Question number	Question	Prompts
1	Can you tell me about your experiences of hearing voices?	
2	Can you tell me how hearing voices has impacted on your relationship with others?	Can you tell me some moments that stand out to you in relation to this? Positive? Negative?
3	Can you tell me how hearing voices has impacted on how others respond to you?	Can you tell me some moments that stand out to you in relation to this? Positive? Negative?
4	Can you tell me how did you make sense of your voices?	What led you to make sense of your voices in this way? Has this changed over time? What resources did you have access to that helped you make sense of your voices?
5	Can you tell me how hearing voices has impacted on your relationship with yourself?	How does this relate to your relationship with others?
6	Can you tell me what voice you think people who hear voices have in society?	How does this impact on you as someone who hears voices?
7	Can you tell me about whether you tell people you hear voices and why?	

Interviews were conducted remotely via phone or video call, as data collection took place over the course of the COVID‐19 pandemic (3 Dec 2020–12 Feb 2021). Interviews lasted between 31 and 92 minutes, were audio‐recorded and transcribed verbatim by the primary researcher. Participants were given the opportunity to take a break or stop the interview at any time. Participants were compensated for their time with a £10 voucher.

### Ethics

The study was approved by the East of Scotland Research Ethics Committee (REC reference: 20/ES/0054; date 02/06/2020) and the Health Research Authority (IRAS number: 278490; date 03/06/2020). All participants gave informed written consent.

### Data analysis

Thematic analysis was used due to its focus on exploring recurrent patterns of meaning and ability to highlight similarities/differences across a sample. Clinical and non‐clinical transcripts were coded simultaneously using the same coding framework, with descriptive themes then generated for each group separately. Descriptive themes surmise multiple similar codes by grouping and summarizing them and remain close to the data. Analytical themes go beyond what is explicitly stated in the data, instead of reflecting the underlying conceptual framework which is used to answer the research question (Braun & Clarke, 2006). Initial coding was completed by the first author (*XX)*, who then generated the initial descriptive themes. These descriptive themes, and the coding framework they related to, were then shared with the rest of the research team. A meeting was then held with the research team within which the initial analytical themes were devised by the team as a whole after reflecting on the descriptive themes, coding framework and data narrative. These themes were then finalized in wording by the first author by drawing on the original transcripts, and then final theme checking was completed by the research team as a whole. Throughout this process, regular supervision of the primary author was held with the final author (*XX*), which allowed regular reflection on the coding and theme forming process, to reduce individual bias.

A primarily inductive approach was used, with some deductive coding drawing on the existing framework of epistemic injustice (Fricker, 2007). Due to the lack of existing research, it was felt that this would allow a data‐led exploratory analysis and would facilitate researchers viewing participants' accounts as portraying their lived reality but reflective of only one specific experience, thus allowing us to identify differences. In addition, thematic analysis does not require a homogenous sample, which we felt was appropriate given the diverse types of people who hear voices.

#### Validity and credibility

Multiple validation methods of findings were used, including: final theme checking by the research team (including an individual with lived experience of voice‐hearing), identification of outliers and keeping of a reflective log.

### Research team and reflexivity

The research team consisted of a trainee clinical psychologist (*XX*), two clinical psychologists (*XX* and *XX*), a psychiatrist (*XX*) and someone who hears voices (*XX*). Most of the researchers approached the analysis from the perspective of professionals working within mental health. *XX* brought expertise in qualitative methods of research. *XX* and *XX* had wide‐ranging experience working clinically and completing research with voice‐hearers, whilst *XX* did not and could bring a more neutral and curious perspective. As a team, we hold a critically reflective perspective on the role of diagnosis and the mental health system, particularly with regard to professional discourses around unusual experiences and pathologizing these. However, we also recognize the majority of the teamwork as mental health professionals and may bring assumptions and biases towards how voices are understood from the clinical part of our identities. Thus, including *XX*'s perspectives was felt to be especially important in order to bring the perspective of a voice‐hearer and mental health service user. Her contributions involved contributing to the design of the study, the study materials and protocol, attending the analysis meetings, inputting on the descriptive and analytic themes and commenting on the final manuscript. Examples of her contributions included emphasizing the importance of not being biased towards negative perceptions of voices and adding a question to the interview schedule (‘Can you tell me about whether you tell people you hear voices and why?’).

## RESULTS

### Participants

Seventeen participants in total took part in the study. Nine non‐clinical and five clinical participants were recruited via non‐NHS routes, and three clinical participants were recruited via the NHS. A further 10 people expressed an interest in the study but did not take part. At this point, the data had a high level of information power and was judged to be sufficient to address the research aim (Malterud et al., 2016).

The clinical group consisted of four women, three men and one participant who choose not to disclose their gender identity. Three participants identified as White British, two as British, one as Asian, one as mixed White‐Black Caribbean and one choose not to disclose their ethnic identity. Five participants were recruited via non‐NHS services, and three were recruited via NHS Early Intervention and Community Mental Health teams. Two participants were aged 18–24, two were aged 25–29, one was aged 40–44, one was aged 55–59, one was aged 70+, and one choose not to disclose their age. All had heard voices for over a year, with voice‐hearing length ranging from 1.5 to 70 years (there was no minimum length of voice‐hearing in the inclusion criteria for participants recruited through NHS services as by definition they had already developed a ‘need for care’).

The non‐clinical group consisted of six women and three men. Two participants identified as White British, three as British, one as Pakistani, one as Swiss, one as Black and one as Indigenous American. Four participants were recruited by contacting special interest groups (spiritualist churches and angelic healing centres), one by snowballing and four via placing an advert with the Society of Psychical Research. One participant was aged 25–29, one was aged 40–44, two were aged 50–54, two were aged 55–59, two were aged 60–64, and one was aged 65–69. All had heard voices for over five years, with voice‐hearing length ranging from 6 to 50 + years.

The participants recruited via the NHS had diagnoses of unspecific non‐organic psychosis (F29.X), first episode psychosis (F29) and differential diagnosis of moderate depressive episode (F321). Two of the participants were taking anti‐psychotics at the time of the study, and they had been known to services between a range of 1–3 years.

On the screening measures, the clinical group had a mode of 4 (clear evidence of voices that occur every day) (range = 4–5), and the non‐clinical group had a mode of 3 (clear evidence of voices; they have occurred at least weekly) (range = 2–4) on the SAPs, with the non‐clinical group having a mode of 3 (no need) (range = 2–3) on item 5 and 9 on the CANSAS‐P. This shows that the non‐clinical group did not have unmet self‐care or psychological needs and thus are very unlikely to be members of the clinical population who have yet to be identified as requiring care from mental health services. Moreover, the overlapping range on the SAPs of both groups indicates similar frequency of voice‐hearing, although the modal average was higher in the clinical group.

### Findings

**Figure 1:**
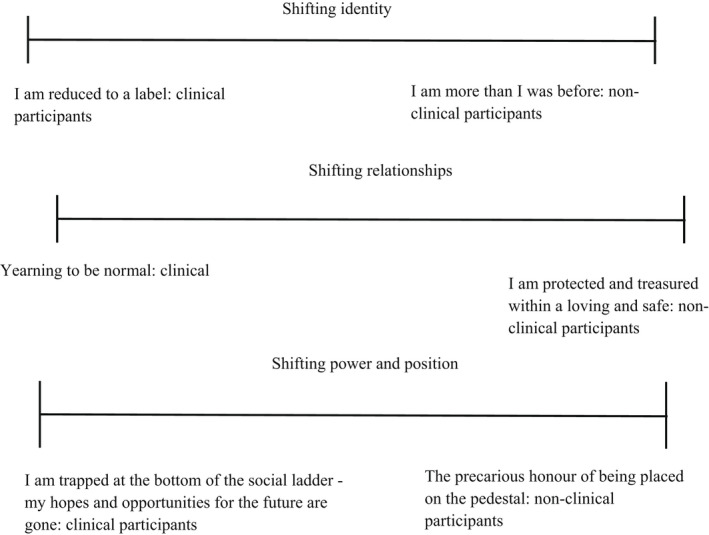
Map of themes including dimensional spectrums of theme pairs

**Figure 2:**
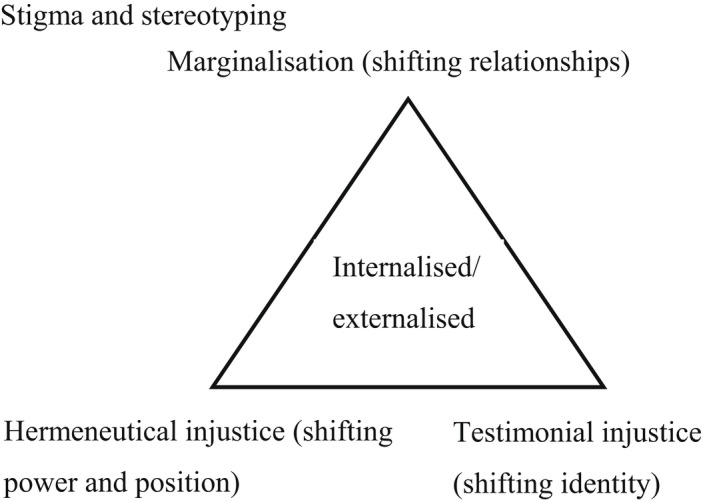
Map of forms of injustice found within the conceptual framework

Overall, a journey of changing identity, relationships and social position within the world was described by those with both clinical and non‐clinical experiences of voice‐hearing. However, whilst themes occurred on a dimensional spectrum (i.e. ‘I am reduced to a label’ vs. ‘I am more than I was before’), there was a clear mirroring of experiences between the two groups reflected in the below themes occurring in pairs. See Table 2 for examples of codes, descriptive themes and analytic themes and Figures 1 and 2 for maps of the conceptual framework.

**Table 2:** Examples of codes, descriptive themes and final analytic themes

Analytical themes	Descriptive themes	Codes	Group
I am reduced to a label	I am reduced to a label Hearing voices changed my relationship with myself I don't like myself anymore The reframing of voice‐hearing from ‘abnormal and shocking’ to ‘normal and natural’ as reducing shame and stigma, and allowing for increased hope of recovery	Being a voice‐hearer means you are limited in what you can do with your life I am limited in the spaces I can occupy within society because of stigma I did not have a choice—lack of information and collaboration within the diagnosis and or treatment process as disempowering voice‐hearers ‘I don't want to be put in a box or seen as a label’—voice‐hearers as reduced to a category—‘they don't see us as individuals’ Mental health as more stigmatized, and therefore seen as less acceptable to discuss and incomparable to, physical health My identity as a ‘voice‐hearer’ is seen as ‘trumping’ pre‐existing skills and identities = my ability to do my job is questioned Others view voice‐hearing as defining of my identity	Clinical
I am more than I was before	Voice‐hearing is just a part of me I am more than I was before Hearing voices changed my relationship with myself	I am a teacher—voice‐hearing as giving me a new part of my identity I am a teacher of voice‐hearing and mediumistic skills—it is my job to guide my students in how to understand their voices, communicated about them and make sense of them	Non‐Clinical
Yearning to be normal	Relationships are more challenging Society views voice‐hearers as dangerous, abnormal and shocking—I am marginalized and stigmatized The voices are my tormentors, torturers and enemies	Voice‐hearing has changed how I relate to people Voice‐hearing has changed the role I hold in social groups = I feel different and like an outsider Voice‐hearing means I am no longer compared to previous or ‘normal’ standards ‘I just want to be normal’—desire to be normal as opposed to an ‘abnormal’ voice‐hearer	Clinical
I am protected and treasured within a loving and safe community	Voice‐hearing as a source of community and connection You are instantly part of the pack The voices are my friends, allies and protectors	Voice‐hearing has not changed how others respond to me Voice‐hearing helps me help others—I can give them information and that is profound Voice‐hearing as a source of connection and community There are pockets of society and communities within which I can be open and feel safe There is a community for voices hearers—but it is a specific and distinct community that is not well accepted by society or open Voice‐hearing allows me to help people Voice‐hearing has allowed me to connect with a new community I am loved and treasured in this community I am loved and treasured by the voices I am viewed as a leader, healer and teacher—I hold an important and influential position in my community because I am a voice‐hearer	Non‐Clinical
I am trapped at the bottom of the social ladder ‐ my hopes and opportunities for the future are gone	Social narratives that voice‐hearing is shameful and abnormal lead to a perception it is ‘not OK’ to discuss voice‐hearing—this undermines voice‐hearers ability to have a voice in society and reinforces feelings of loneliness and internalized stigma I am scared to disclose—I know I will be judged and people will make incorrect assumptions about me based on stigmatizing stereotypes, and I fear how this will change how they see and respond to me I am marginalized and ostracized I am rejected and alone I am at the bottom of the social ladder	I am marginalized within society because others make incorrect assumptions about me due to prevalent stigmatizing narratives about voice‐hearers I am ostracized—voice‐hearing leading to a change in my social status and who would engage with me I am ostracized and people fear me due to stigmatizing and stereotyped assumptions about voice‐hearers I am part of a marginalized group that I want to advocate for ‘I am reduced to a label’—being a voice‐hearer reduces my identity to being a voice‐hearer and ‘ill’ or ‘crazy’ Negative impact on your sense of self from the stigmatizing views of others—internalized stigma Negative stereotypes and social narratives and reactions reinforcing negative perceptions I hold of myself due to hearing voices Mental health and voice‐hearing as indicative of a personal failing, unlike physical health—therefore should be hidden and shows you ‘can't’ occupy particular roles in society	Clinical
The precarious honour of being placed on the pedestal	Being a voice‐hearer leads me to be ‘put on a pedestal’—I am looked up to I am a teacher, leader and healer I am a community leader I am looked up to and revered	I am a teacher—voice‐hearing as giving me a new part of my identity I am a teacher of voice‐hearing and mediumistic skills—it is my job to guide my students in how to understand their voices, communicated about them and make sense of them I am viewed as a leader, healer and teacher—I hold an important and influential position in my community because I am a voice‐hearer I give information to others using my gifts—this is a profound way to heal and help	Non‐Clinical
Not everyone finds their lighthouse	I cannot access the concepts needed for me to make meaningful sense of my voices—as such, the experience is more overwhelming, I cannot put my experience into words and I am led to make sense of them in more catastrophising and frightening ways (hermeneutical injustice) I was empowered to make sense of my experiences by being given access to concepts and language which helped me make sense of my experiences in a meaningful way ‘We are all in the same boat’—relationships with people with experience of voice‐hearing allows you to access new concepts, feel seen and understood, and therefore better understand your experience	Ability to make sense of the voices as impacting how overwhelming the voices are Clarity of understanding impacted by the ability to make sense of the voices Concepts needed to make sense of voice‐hearing are not readily available generally within society Difficulty making sense of voices makes it hard to put experiences into words Generally in society voice‐hearers do not have access to ideas which enable them to make sense of their experience—making it more overwhelming, confusing and harder to put the experience into words Being given access to new ideas as empowering sense‐making by helping me put words to my experience Having a language to express my experience is key to mine and others’ understanding Having more access to concepts which facilitated sense‐making would enable people to feel more confident to seek help ‘Getting the message out there’—promoting voice‐hearers’ voices allows for alternative social narratives to be formed that help voice‐hearers feel accepted, and to understand their experience (in a non‐medicalised form) If I cannot express my experiences in words then others cannot understand that part of my life and it is ‘hidden’ Inability to make sense of the voices in a useful way as making me ‘more ill’ Inability to put the experience of voice‐hearing into words means others cannot understand I can only make meaningful sense of the voices when I am empowered to access relevant ideas	Shared
I am cast as the unreliable narrator of my own experience	Voice‐hearing identity leading others to believe the person less and them be seen as ‘less credible’ Voice‐hearers leading them to be dismissed regarding their own lived experience Voice‐hearers are not believed about their voices or other things they give testimony about	I am believed less as I am a voice‐hearer Others question what the voices as saying about them—they are trust in me is diminished because I'm a voice‐hearer I am discriminated against because of voice‐hearing I am dismissed—my opinions are seen as less important and valid as a voice‐hearer If I come across as ill, others will see me as less credible and give less weight to what I say My opinions are given less weight as I am a voice‐hearer My perception and understanding of my own symptoms is viewed as less valid because I am a voice‐hearer and therefore ‘ill’ Narrative that voice‐hearers should be believed less or are less credible as further feeding into marginalization and their voices not being heard in society My views on things over than voices are dismissed because I am a voice‐hearer (global testimonial injustice)	Shared

#### Changing identity thematic pair

1. I am reduced to a label: clinical group themeClinical participants reflected a strong sense that their identity had been obliterated and replaced by their identity as a voice‐hearer. Participants found this cascaded from their past, into their present and the future, eradicating previous achievements and skills, limiting future opportunities and relationships and defining how they were seen by others and saw themselves in the present. Social narratives of voice‐hearers as violent, dangerous, abnormal and untrustworthy dominated social interactions and their new sense of self, with participants experiencing high levels of stigma which, by being internalized, shaped how they saw themselves. This stood at odds with the experiences of the non‐clinical participants, who experienced their voice‐hearing as adding to their identity in a positive way without overriding their existing identities (see non‐clinical group theme):
  I think generally they are perceived as a little bit sort of… words almost psycho they're perceived as a bit quirky or weird or… yeah um I think people are a bit scared about it almost like yeah people are almost a bit scared about it and yeah that definitely had an impact on me sort of saying about it and opening up about it (C6)
2. I am more than I was before: non‐clinical group themeNon‐clinical participants reflected that voice‐hearing had positively added to their identity, giving them a unique skill, a special power, a sense of purpose and for many a passion and career. They characterized voice‐hearing as just a part of who they are, which added to, but did not define, their identity or relationship with themselves. For many, the voices formed friends who acted as their confidants and protectors and added colour and richness to their lives:
  it feels like that voice is more than just a voice it's like… it sometimes feels like this voice is hugging me or embraces me or… yeah… it's like I'm having kind of a relationship with that voice it's like having a best friend or something (laughs) (NC4)

#### Changing relationships thematic pair

1. Yearning to be normal: clinical group themeParticipants reflected that their label of voice‐hearer led them to be ostracized, rejected and marginalized within society. They noted that their experiences ran counter to what was seen as ‘normal’, resulting in voice‐hearing being perceived as shocking and abnormal. This left them with a profound sense of being societal outsiders who existed on the fringes of normal life. They reported a deep yearning to be normal and able to re‐engage with society:
  I suppose I… I didn't want to be viewed as insane or mad or weird or cause no one wants to be perceived as weird or odd or sort of societally unacceptable um they they want to sort of fit in and be accepted and it's quite hard to think that potentially you might not … (C6)
2. I am protected and treasured within a loving and safe community: non‐clinical group themeParticipants reported that the voices increased their social contact, integrating them into a loving and accepting community where they felt cherished, treasured and loved. For them, the voices increased their social connectedness, inviting new relationships with other voice‐hearers, strengthening existing relationships and also adding the new relationships with the voices themselves. However, there was a strong sense that whilst they were safe in the harbour of this community, the community was rejected by mainstream society and thus there was danger when they left this specific, protected harbour. This contrasted with the experience of the clinical participants who had no safe harbour to escape social rejection and ostracization, thus losing old relationships and the ability to form new ones due to their identity as a voice‐hearer (see clinical group theme):
  I think you have an innate connection with people who are like‐minded … you know you are drawn to social groups with the same‐minded … you tend to know people who are soul mates on your journey you know (NC5)

#### Changing power and position thematic group

1. I am trapped at the bottom of the social ladder—my hopes and opportunities for the future are gone: clinical group themeParticipants reflected a significant sense that they were now at the bottom of the social ladder, and moreover that they were stuck there as the rungs they could have climbed to escape had been removed. There was a profound sense of hopelessness amongst the clinical participants related to concerns that their hopes and opportunities in the future had been washed away by the social response to their identity as a voice‐hearer. This in turn appeared to manifest as a deep sense of grief for the person they used to be and who they could have become. This stood at odds with the non‐clinical participants, who found voice‐hearing placed them in elevated positions of power and influence, looked up to and at points revered, if only within their limited safe community (see non‐clinical theme):
  but as… as for finding employment I don't suppose anyone would touch… touch me with a barge pole I don't know how… virtually unemployable I would imagine (C2)
  I don't tend to tell people but it… it's really hard to describe it's like their energy towards you changes … I think they've learnt a lot of this behaviour from tv …I have had the question well if you hear voices why aren't you in a mental hospital and that's quite upsetting to me cause like that's not that's you know that's not the place for everyone (C7)
2. The precarious honour of being placed on the pedestal: non‐clinical group themeNon‐clinical participants reflected that becoming a voice‐hearer led to an elevation in their social status, resulting in others placing them on a pedestal and viewing them as leaders, teachers and healers. They described others coming to them for wisdom, guidance and healing, particularly the mediums who view voice‐hearing as a gift that allows them to help others. However, there was a sense this was a precarious honour, with those outside of their community rejecting and misunderstanding them, meaning they could be knocked from the pedestal at any time:
  when they're learning they tend to think you have some sort of superhuman power… um… so… so very often you are put on a bit of a pedestal and then my job is then to encourage clients and students that everything I have is actually very normal (NC1)
3. Not everyone finds their lighthouse: shared themeAcross both groups, a recurrent theme was the impossibility of making sense of voice‐hearing when it first occurred due to the complete absence of shared, societal concepts which allowing for meaningful sense‐making (hermeneutical injustice). Voice‐hearers felt forced into using the only available concepts to put words to their experience and thus had to draw on a medicalised approach which they felt at odds with and pressured into using. However, some voice‐hearers went on to find a community, such as a spiritualist church or the hearing voices network, where they met other voice‐hearers and were given concepts, skills and a language which allowed them to make sense of what was happening. These safe communities served as a sort of lighthouse, guiding them towards safe waters and protecting against hermeneutical injustice. Thus, the ability to find some sort of community, even if they were rejected by mainstream society, was crucial to recovery for both groups (see the clinical and non‐clinical group themes for this thematic group):
  you can kind of start a sentence and then kind of forget what you're saying and someone's like yeah I know what you mean you mean this and I'm like yeah I mean that … there was something super profound about… about that experience… its just like you're immediately in a pack … I often feel alone in my experience… but that aloneness really kind of shifted … you know you're a fisherman and … like you're alone in your experience of fishing but you know there are other fishman who are there you know that if you fell off that they would kind of come and help you (C1)
4. I am cast as the unreliable narrator of my own experience: shared themeFinally, most participants reflected on being cast as an unreliable narrator due to their voice‐hearing experiences, with others tending to dismiss them as not to be believed and less credible due to their voice‐hearing (testimonial injustice). This included disbelief of their symptoms and own framework of understanding around this, as well as a more general disbelief of anything they said due to them being perceived as untrustworthy as they heard voices. This led the participants in turn to become less certain in themselves, resulting in some actually starting to question their own thoughts, feelings and emotions due to this testimonial injustice they were facing:
  people are often dismissed in society and you know and there's a lot of it's a lot for people to get their heads around especially like with media and um the label schizophrenia and with particular connotations um then you know then people have said to me I have this label schizophrenia and then I look in the paper and this is what this is what it says you know and that in itself is like well but yeah the media's the media (C2)
  I think it is a worry I think it is a worry that people don't don't believe what I feel don't think that it really exists (C8)

## DISCUSSION

This paper describes a qualitative study exploring experiences of voice‐hearing amongst 17 clinical and non‐clinical voice‐hearers. Two pairs of themes were constructed from semi‐structured interviews related to (i) identity and (ii) relationships and one group theme related to (iii) power and position.

The findings reflect existing research, which has shown voice‐hearers are at risk of both testimonial and hermeneutical injustice (Sanati & Kyratsous, 2015; McCarthy‐Jones et al., 2013), and differences in social responses to clinical and non‐clinical voice‐hearers (Heriot‐Maitland et al., 2012). In addition, findings cohere with research which has shown voice‐hearers experience high levels of stigma, and for some this can become internalized, impacting on self‐esteem and recovery (Burke et al., 2016; Wood et al., 2017a). Interestingly, findings in this study suggested whilst both groups experienced stigma, for non‐clinical voice‐hearers the existence of a ‘safe harbour’ community where epistemic injustice was not present prevented this stigma from becoming internalized and impacting on individual's self‐image. We may suggest this is because this community provided a space where individuals were socially connected and held ongoing social capital due to being viewed as epistemic equals or, at times, sources of knowledge. This aligns with research which has shown these to be factors in mitigating internalized stigma (Pyle et al., 2018). This may suggest that exploring an individual's experience of epistemic injustice can allow greater insight into the process of stigma becoming internalized, which studies have highlighted is a key mediator between stigma, self‐esteem and recovery (Vass, Sitko, West & Bentall, 2017).

In terms of the strengths and limitations of the study, the main strength is inclusion of a person with lived experience as part of research team. In addition, whilst previous literature in this area was mainly theoretical, this paper presents empirical data on the lived experience of people who hear voices, significantly adding to the existing evidence‐base (Lee et al., 2019; Tate, 2019). In terms of limitations, coding was completed by the first author alone, increasing the risk of bias. Whilst the sample displayed some diversity in terms of age, gender and ethnicity, the participants were predominantly white, from Western societies and most of the non‐clinical group were part of the same sub‐society. Thus, the results may not be generalizable to other ethnic groups or cultures not represented in the sample. This is particularly important given wide cultural differences in how voices, and mental health difficulties, are conceptualized in general. However, generalisability is not often the goal of qualitative research, and the concept of information power may be more appropriate to draw on. As discussed previously, given the rich data set collected, the study had a high level of information power.

### Clinical implications and future research

This paper shows the profound impact epistemic injustice can have on voice‐hearers, showing that tackling this form of discrimination should be an important part of any mental health service's approach with this population. Thus, this paper serves as a challenge to services and professionals to adapt their practice to ensure they are dismantling, not enacting, epistemic injustice within their interactions with service users. Findings in this paper highlight that services need to provide a community for voice‐hearers where they are epistemically empowered. We suggest that to do this is to do more than offer psychosocial interventions within a medicalised system, but rather adapt their system functioning to provide a ‘safe harbour’ where clients are believed, given access to a variety of sense‐making frameworks and treated as epistemic equals. To reach a greater sense of epistemic equality, services will need to place greater emphasis on promoting and listening to the voices of voice‐hearers. This may involve using more peer‐support approaches, recruiting experts by experience and involving them in service design. There is also a need for services to tackle stigma and marginalization, including internalized stigma, which may be further disempowering voice‐hearers and contributing to distress. Existing research has suggested interventions can be helpful in addressing internalized stigma and improving self‐esteem within this population (Wood, Byrne, Varese, & Morrison, 2016; Best et al., 2018). Interestingly, these interventions tend to be focussed on working with the individual themselves, using cognitive restructuring or social skills training to address the impact of stigma. We may argue this study has shown the wider systemic issues, such as power imbalances and structures that promote epistemic inequality, that can contribute to individuals' experiences of stigma. Thus, something we may take away from this is the need for services to tackle stigma not only on an individual, but societal level, such as through anti‐stigma campaigns.

Overall, we would argue that this study has shown the conceptual framework of epistemic injustice can add to existing understandings of voice‐related distress, stigma and marginalization, by providing a language to describe patterns of discrimination related to epistemic power imbalances and maintain stigma at a systemic and interpersonal level. Findings from this initial study have shown this framework is relevant and applicable to the experiences of those who hear voices. More research is needed to build on these findings to see whether this framework has applications understanding the experiences of other groups with mental health difficulties. Moreover, research is needed to operationalize ‘epistemic injustice’ to allow professionals to draw on it as a tool to assess practice at an individual and service‐wide level. Following this, it would be interesting to assess how epistemic injustice relates to other concepts of interest in this area, such as internalized stigma, shame and distress. Finally, interventions to promote epistemic equality within services, such as looking to include peer‐support workers within initial assessment or establishing a practice that all service users have a consultation with an expert by experience, could also be explored within future research.

## CONCLUSION

Voice‐hearers, regardless of whether they experienced voice‐related distress or not, were subject to widespread experiences of epistemic injustice, which were seen to arise from entrenched mental health stigma in society. Some voice‐hearers described significant benefit from having access to a community (‘safe harbour’) where they were loved, believed and helped to make meaningful sense of their experiences. These insights could help improve care for people receiving mental health care for distressing voices, who sometimes experienced psychiatric services as perpetuating epistemic injustice.

## CONFLICT OF INTEREST

All authors have no conflict of interest to declare.

## AUTHOR CONTRIBUTIONS

OH and PJ led the design of the study. OH conducted the qualitative interviews and led the analysis, with all authors contributing to data interpretation. OH wrote the initial draft of the manuscript, and all authors contributed to revising and finalising the manuscript, and read and approved the final version for submission.

## Data Availability

Data for this study are not available, due to the sensitive nature of the data set. The study is qualitative, and thus, the data consist of interview transcripts, which are of a sensitive and confidential nature. In line with standard practice, these are not publicly available.

## References

[bjc12368-bib-0001] Anderson, N. (1984). Scale for the assessment of positive symptoms (SAPS). University of Iowa Press.

[bjc12368-bib-0002] Baumeister, D. , Sedgwick, O. , Howes, O. , & Peters, E. (2017). Auditory verbal hallucinations and continuum models of psychosis: A systematic review of the healthy voice‐hearer literature. Clinical Psychology Review, 51, 125–141. 27866082 10.1016/j.cpr.2016.10.010 PMC5240854

[bjc12368-bib-0003] Bebbington, P. , & Nayani, T. (1995). The psychosis screening questionnaire. International Journal of Methods in Psychiatry Research, 5(1), 11–19.

[bjc12368-bib-0004] Best, M. W. , Grossman, M. , Milanovic, M. , Renaud, S. , & Bowie, C. R. (2018). Be outspoken and overcome stigmatizing thoughts (BOOST): A group treatment for internalized stigma in first‐episode psychosis. Psychosis, 10(3), 187–197.

[bjc12368-bib-0005] Braun, V. , & Clarke, V. (2006). Using thematic analysis in psychology. Qualitative Research in Psychology, 3(2), 77–101.

[bjc12368-bib-0006] Braun, V. , & Clarke, V. (2013). Successful qualitative research: a practice guide for beginners. Sage Publications.

[bjc12368-bib-0008] Burke, E. , Wood, L. , Zabel, E. , Clark, A. , & Morrison, A. P. (2016). Experiences of stigma in psychosis: A qualitative analysis of service users' perspectives. Psychosis, 8(2), 130–142.

[bjc12368-bib-0009] Carel, H. , & Kidd, I. J. (2014). Epistemic injustice in healthcare: A philosophical analysis. Medicine Health Care and Philosophy, 17(4), 529–540. 10.1007/s11019-014-9560-2 24740808

[bjc12368-bib-0012] Crichton, P. , Carel, H. , & Kidd, I. J. (2017). Epistemic injustice in psychiatry. *Bjpsych* . Bulletin, 41(2), 65–70. 10.1192/pb.bp.115.050682 PMC5376720 28400962

[bjc12368-bib-0013] Fricker, M. (2007). Epistemic injustice: Power and the ethics of knowing. Oxford University Press.

[bjc12368-bib-0016] Heriot‐Maitland, C. , Knight, M. , & Peters, E. (2012). A qualitative comparison of psychotic‐like phenomena in clinical and non‐clinical populations. British Journal of Clinical Psychology, 51, 37–53. 10.1111/j.2044-8260.2011.02011.x 22268540

[bjc12368-bib-0017] Houlders, J. W. , Bortolotti, L. , & Broome, M. R. (2021). Threats to epistemic agency in young people with unusual experiences and beliefs. Synthese, 199, 7689–7704. 10.1007/s11229-021-03133-4 34970007 PMC8668839

[bjc12368-bib-0018] Huggett, C. , Birtel, M. D. , Awenat, Y. F. , Fleming, P. , Wilkes, S. , Williams, S. , & Haddock, G. (2018). A qualitative study: Experiences of stigma by people with mental health problems. Psychology and Psychotherapy: Theory, Research and Practice, 91(3), 380–397. 10.1111/papt.12167 29345416

[bjc12368-bib-0019] Johns, L. C. , Kompus, K. , Connell, M. , Humpston, C. , Lincoln, T. M. , Longden, E. , Pretim, A. , Alderson‐Day, B. , Badcock, J. C. , Cella, M. , Fernyhough, C. , McCarthy‐Jones, S. , Peters, E. , Raballo, A. , Scott, J. , Siddi, S. , & Larøi, F. (2014). Auditory verbal hallucinations in persons with and without a need for care. Schizophrenia Bulletin, 40(Suppl_4), S255–S264. 24936085 10.1093/schbul/sbu005 PMC4141313

[bjc12368-bib-0020] Kidd, I. J. , & Carel, H. (2017). Epistemic injustice and Illness. Journal of Applied Philosophy, 34(2), 172–190. 10.1111/japp.12172 28303075 PMC5324700

[bjc12368-bib-0021] Lee, E. , Tsang, A. K. T. , Bogo, M. , Johnstone, M. , Herschman, J. , & Ryan, M. (2019). Honoring the voice of the client in clinical social work practice: Negotiating with epistemic injustice. Social Work, 64(1), 29–38. 10.1093/sw/swy050 30364977

[bjc12368-bib-0022] Linscott, R. J. , & van Os, J. (2010). Systematic reviews of categorical versus continuum models in psychosis: Evidence for discontinuous subpopulations underlying a psychometric continuum. Implications for DSM‐V, DSM‐VI, and DSM‐VII. Annual Review of Clinical Psychology, 6, 391–419. 10.1146/annurev.clinpsy.032408.153506 20192792

[bjc12368-bib-0023] Malterud, K. , Siersma, V. D. , & Guassora, A. D. (2016). Sample size in qualitative interview studies: Guided by information power. Qualitative Health Research, 26(13), 1753–1760. 26613970 10.1177/1049732315617444

[bjc12368-bib-0025] McCarthy‐Jones, S. , Marriott, M. , Knowles, R. , Rowse, G. , & Thompson, A. R. (2013). What is psychosis? A meta‐synthesis of inductive qualitative studies exploring the experience of psychosis. Psychosis, 5(1), 1–16.

[bjc12368-bib-0027] Oakland, L. , & Berry, K. (2015). "Lifting the veil": A qualitative analysis of experiences in Hearing Voices Network groups. Psychosis‐Psychological Social and Integrative Approaches, 7(2), 119–129. 10.1080/17522439.2014.937451

[bjc12368-bib-0028] Peters, E. , Ward, T. , Jackson, M. , Morgan, C. , Charalambides, M. , McGuire, P. , Woodruff, P. , Jacobsen, P. , Chadwick, P. , & Garety, P. A. (2016). Clinical, socio‐demographic and psychological characteristics in individuals with persistent psychotic experiences with and without a "need for care". World Psychiatry, 15(1), 41–52. 10.1002/wps.20301 26833608 PMC4780307

[bjc12368-bib-0029] Pyle, M. , Pilling, S. , Machin, K. , Allende‐Cullen, G. , & Morrison, A. P. (2018). Peer support for internalised stigma experienced by people with psychosis: Rationale and recommendations. Psychosis, 10(2), 146–152.

[bjc12368-bib-0030] Sanati, A. , & Kyratsous, M. (2015). Epistemic injustice in assessment of delusions. Journal of Evaluation in Clinical Practice, 21(3), 479–485. 10.1111/jep.12347 25828924

[bjc12368-bib-0040] Trauer, T. , Tobias, G. , & Slade, M. (2008). Development and evaluation of a patient‐rated version of the camberwell assessment of need short appraisal schedule (CANSAS‐P). Community Ment Health J, 44, 113–123. 10.1007/s10597-007-9101-z 17701455

[bjc12368-bib-0032] Tate, A. J. M. (2019). Contributory injustice in psychiatry. Journal of Medical Ethics, 45(2), 97–100. 10.1136/medethics-2018-104761 30337450 PMC6388905

[bjc12368-bib-0034] Vass, V. , Sitko, K. , West, S. , & Bentall, R. P. (2017). How stigma gets under the skin: The role of stigma, self‐stigma and self‐esteem in subjective recovery from psychosis. Psychosis, 9(3), 235–244.

[bjc12368-bib-0035] Wood, L. , Byrne, R. , Varese, F. , & Morrison, A. P. (2016). Psychosocial interventions for internalised stigma in people with a schizophrenia‐spectrum diagnosis: A systematic narrative synthesis and meta‐analysis. Schizophrenia Research, 176(2–3), 291–303. 27256518 10.1016/j.schres.2016.05.001

[bjc12368-bib-0036] Wood, L. , Byrne, R. , Burke, E. , Enache, G. , & Morrison, A. P. (2017a). The impact of stigma on emotional distress and recovery from psychosis: The mediatory role of internalised shame and self‐esteem. Psychiatry Research, 255, 94–100. 28531822 10.1016/j.psychres.2017.05.016

[bjc12368-bib-0037] Wood, L. , Byrne, R. , & Morrison, A. P. (2017b). An integrative cognitive model of internalized stigma in psychosis. Behavioural and Cognitive Psychotherapy, 45(6), 545–560. 28488561 10.1017/S1352465817000224

[bjc12368-bib-0038] World Health Organization(WHO) . (1993). The ICD‐10 classification of mental and behavioural disorders. World Health Organization.

